# RETRACTED: Bakshi et al. Crocin Inhibits Angiogenesis and Metastasis in Colon Cancer via TNF-α/NF-kB/VEGF Pathways. *Cells* 2022, *11*, 1502

**DOI:** 10.3390/cells15131197

**Published:** 2026-07-01

**Authors:** Hamid A. Bakshi, Gerry A. Quinn, Mohamed M. Nasef, Vijay Mishra, Alaa A. A. Aljabali, Mohamed El-Tanani, Ángel Serrano-Aroca, Mateus Webba Da Silva, Paul A. McCarron, Murtaza M. Tambuwala

**Affiliations:** 1 School of Pharmacy and Pharmaceutical Sciences, Ulster University, Coleraine BT52 1SA, UK; mm.webba-da-silva@ulster.ac.uk (M.W.D.S.); p.mccarron@ulster.ac.uk (P.A.M.); 2 School of Biomedical Sciences, Ulster University, Coleraine BT52 1SA, UK; g.quinn@ulster.ac.uk; 3 Department of Pharmacy, School of Applied Sciences, University of Huddersfield, Huddersfield HD1 3DH, UK; mohamednasef103@gmail.com; 4 School of Pharmaceutical Sciences, Lovely Professional University, Phagwara 144411, Punjab, India; vijaymishra2@gmail.com; 5 Department of Pharmaceutics and Pharmaceutical Technology, Yarmouk University, Irbid 566, Jordan; alaaj@yu.edu.jo; 6 Pharmacological and Diagnostic Research Centre, Faculty of Pharmacy, Al-Ahliyya Amman University, Amman 19328, Jordan; m.tanani@ammanu.edu.jo; 7 Biomaterials and Bioengineering Lab, Centro de Investigación Traslacional San Alberto Magno, Universidad Católica de Valencia San Vicente Mártir, 46001 Valencia, Spain; angel.serrano@ucv.es

The journal retracts the article titled, “Crocin Inhibits Angiogenesis and Metastasis in Colon Cancer via TNF-α/NF-kB/VEGF Pathways” [1], cited above.

Following publication, concerns were brought to the attention of the Editorial Office regarding the integrity of the images presented in this publication [1].

In accordance with the journal’s standard procedures, the Editorial Office and Editorial Board conducted an investigation that identified indications of inappropriate image editing of Figure 2B, an overlap between Figure 3A,B, and overlap between Figure 5A,B, all showing different experimental conditions published in [1]. An investigation also confirmed an overlap between Figure 3A published in [1] and Figure 5B published earlier in [2], showing different experimental conditions, as well as an overlap between Figure 4C published in [1] and Figure 4B published earlier in [3] by a different author group. Although the authors cooperated with the investigation, the explanations and supporting materials provided were not deemed sufficient by the Editorial Board to resolve the identified concerns. As a consequence, the Editorial Board lost confidence in the reliability of the overall findings and has decided to retract this publication in accordance with MDPI’s retraction policy.

This retraction was approved by the Editor-in-Chief of the journal *Cells*.

The authors have been informed of this retraction. The 2nd (G.A.Q.), the 4th (V.M.), and the 5th (A.A.A.) authors disagreed with the retraction, the corresponding author (M.M.T.) agreed with the retraction, while the rest of the authors did not provide a comment on the decision.
